# A Criticism of Dr. Holmes

**Published:** 1888-12

**Authors:** J. T. Kent


### A CRITICISM OF DR. HOLMES.

EDITORS HOMŒOPATHIC PHYSICIAN:—On page 602, this year, my friend Holmes relates a perfectly simple Veratrum case and cure; a case that a recent graduate would not fail to recognize at a glance. He further reflects upon experience when he states that he did not have his "library" with him, and he had loaned out his wheelbarrow. If Dr. Holmes had told us what he would have given or done had he found a case of sickness that presented symptoms entirely unknown to him, I would refrain from asking him to please come out again frankly and state just what he would have done. I believe that Dr. Holmes is honest, and therefore believe that he would have been sorry he had loaned his wheelbarrow, and sorry he had not brought his repertory. Dr. Holmes would have us believe that he thinks that doctors carry their repertory simply to make a show, simply to look for such simple cases as he reports. I do not know a member of the International Hahnemannian Association that would need a repertory for so simply a case as the Veratrum case. Perhaps Dr. Holmes offers this as a stumper—a case that would puzzle the honorable members of the International Hahnemannian Association. If Dr. Holmes offers this case to show his own erudition, and the full extent of it, he has succeeded, but if he has offered it to show that the repertory is not a valuable life-saving plan, he has failed.

He intimates that his "rule of practice" is to give a medicine high, but if his "rule of practice" is based upon the same reasoning as his rule of leaving his library at home (because a low potency would be so heavy to carry in a hurry), we presume his potency, therefore, was very high.

He gives six powders, but does not say how much better six doses would be than one; therefore we infer that six powders, one every half-hour, must be also a "rule of practice."

He says: "I consider this a desperate case, as several such had died under old-school treatment."

"As several such had died under old-school treatment" was his reason for thinking it a desperate case, and the only reason for thinking it a desperate case, we have no evidence that the prescription cured. He may have simply lived because he did not get old-school treatment.

"In cases calling for immediate action, it seems to me a risky piece of work to either take out a library at the bedside or to go back to one's office to study it up."

We therefore infer Dr. Holmes thinks it not risky to stay at the bedside of a violent sickness, even if one knows not the remedy for this sickness. What will Dr. Holmes do in the absence of knowing what to do that is right? Will he look on and let the patient die? Will he guess at one or several remedies? Will he break the law and give allopathic drugs? or what will he do? Does Dr. Holmes mean to have us infer that he, a young man, has so much wisdom and materia medica in his head that he is never puzzled? He attempted to convince us of that at Niagara, but made a signal failure.

"I have not, as a rule, been able to find just what I wanted when I was in a hurry." He means that he is not accustomed to the repertory so that he can find what he wants in a hurry. This is a criminal confession for a professed follower of Hahnemann. The confession means negligence or laziness when human life is at stake.

"Let those use their books who want or need them." By this Dr. Holmes says, in substance, that he does not want books and does not need them. This is an astonishing statement. I would like to study materia medica under Dr. Holmes.

J. T. KENT.

